# REV-ERB is essential in cardiac fibroblasts homeostasis

**DOI:** 10.3389/fphar.2022.899628

**Published:** 2022-10-31

**Authors:** Xiaokang Luo, Shiyang Song, Lei Qi, Chih-Liang Tien, Hui Li, Weiyi Xu, Theodore Lemuel Mathuram, Thomas Burris, Yuanbiao Zhao, Zheng Sun, Lilei Zhang

**Affiliations:** ^1^ Department of Molecular and Human Genetics, Baylor College of Medicine, Houston, TX, United States; ^2^ Department of Medicine, Division of Diabetes, Endocrinology and Metabolism, Baylor College of Medicine, Houston, TX, United States; ^3^ Genetics Institute, University of Florida, Gainesville, FL, United States; ^4^ Department of Molecular and Cellular Biology, Baylor College of Medicine, Houston, TX, United States

**Keywords:** REV-ERB, fibroblast activation, SR9009, cardiac fibroblasts, TGFβ

## Abstract

REV-ERB agonists have shown antifibrotic effects in the heart and other organs. The function of REV-ERB in the cardiac fibroblasts remains unstudied. Here, we characterize the functional difference of REV-ERB in mouse embryonic fibroblasts and cardiac fibroblasts using genetic deletion of REV-ERBα and *ß in vitro*. We show that REV-ERB α/β double deleted cardiac fibroblasts have reduced viability and proliferation, but increased migration and myofibroblasts activation. Thus, REV-ERB α/β has essential cell-autonomous role in cardiac fibroblasts in maintaining them in a healthy, quiescent state. We also show that existing REV-ERB agonist SR9009 strongly suppresses cardiac fibroblasts activation but in a REV-ERB-independent manner highlighting the need to develop novel REV-ERB agonists for treating cardiac fibrosis.

## Introduction

The mammalian behavioral and physiological processes follow a 24-h cycle. While the central clock exists in the suprachiasmatic nucleus (SCN) of the brain, the molecular circadian clock is ubiquitously expressed in almost all cell types throughout the body and can operate as peripheral clocks independently of the SCN central clock. Cell type-specific deletion of the clock genes in peripheral tissues has provided critical insights into the function of the peripheral clocks. Cardiomyocyte-specific deletion of the core clock gene BMAL1 show age-associated dilated cardiomyopathy (Lefta et al., 2012; Young et al., 2014; Ingle et al., 2015). Nuclear receptor REV-ERBα and *ß* are encoded by *Nr1d1* and *Nr1d2* genes, respectively. REV-ERBs act as transcription suppressors of the second feedback loop in the core clock (Takahashi, 2017). We and others have recently reported that double deletion of REV-ERBα/β in the cardiomyocytes leads to progressive dilated cardiomyopathy and lethal heart failure, suggesting a critical function of REV-ERB in the cardiomyocytes (Dierickx et al., 2022; Song et al., 2022).

Cardiomyocytes constitute ∼30–40% of the cells in the heart (Pinto et al., 2016; Zhou and Pu, 2016). Many other cell types in the heart have critical functions in cardiac physiology and disease pathogenesis. Cardiac fibroblasts (CFs) activation by various signaling pathways, including the TGF-β pathway, results in cardiac fibrosis and is a well-recognized etiological factor in ischemic heart disease, hypertensive heart disease, hypertrophic cardiomyopathy, and dilated cardiomyopathy (Frohlich, 2001; Jellis et al., 2010; Travers et al., 2016; Disertori et al., 2017). The role of REV-ERB in CFs has not been investigated.

SR9009 is a REV-ERB agonist with various health benefits in different experimental systems (Banerjee et al., 2014; Zhang et al., 2017; Gonzalez-Fernandez et al., 2018; Griffett et al., 2020; Wolff et al., 2020). SR9009 is cardioprotective in myocardial infarction (MI) mice models (Stujanna et al., 2017; Reitz et al., 2019). We have shown that SR9009 can protect the heart in a pressure-overload model by preserving cardiac function, reducing cardiac fibrosis, and limiting pathological remodeling (Zhang et al., 2017). It is unknown whether the reduced cardiac fibrosis is due to reduced cardiac injury *via* a cross-talk between cardiomyocytes and CFs (Manabe et al., 2002; Kakkar and Lee, 2010) or whether SR9009 has a direct effect on CFs.

REV-ERB likely plays a role in the fibroblasts because SR9009 reduces acute cigarette smoke-induced inflammatory response and abnormal epithelial-to-mesenchymal transition in the lung (Wang et al., 2021). SR9009 also suppresses hepatic fibrosis in a non-alcoholic steatotic hepatitis murine model (Griffett et al., 2020). Another REV-ERB agonist GSK4112 inhibits myofibroblasts activation in idiopathic pulmonary fibrosis (IPF) fibroblasts (Cunningham et al., 2020). However, SR9009 has REV-ERB–independent effects in the mouse hepatocytes, embryonic stem cells and cardiomyocytes (Li et al., 2022) (Dierickx et al., 2019). Therefore, it is unclear whether REV-ERB has a cell-autonomous function in fibroblasts and whether the effect of SR9009 in fibroblasts is dependent on REV-ERB. Here, we use genetic approaches to investigate the function of REV-ERB in two different fibroblastic cell types, mouse embryonic fibroblasts (MEFs) and CFs. We also addressed the REV-ERB dependency of SR9009 in those fibroblasts.

## Materials and methods

### Mice

REV-ERBα and *ß* floxed mice were previously described (Rev-erbα^loxP^ (*Nr1d1* ^tm1.2Rev^, MGI ID 5426700) and Rev-erbβ^loxP^ (*Nr1d2* ^tm1.1Rev^, MGI ID 5426699) (Song et al., 2022). They were crossed to generate the double floxed mouse line (*Nr1d1/2* ^*fl/fl*^). The exon three and exon four of *Nr1d1* were floxed, which will lead to an in-frame deletion of the DNA binding domain upon Cre recombinase cleavage (Cho et al., 2012). The exon three of *Nr1d2* was floxed, which leads to a frame-shift deletion and nonsense-mediated decay of the transcript upon Cre recombinase cleavage (Cho et al., 2012). All the animal procedures were approved by the Institutional Animal Care and Use Committee at Baylor College of Medicine.

### Fibroblasts cultures and treatments

MEFs were isolated at E13.5 as previously described (Durkin et al., 2013). CFs were isolated from 8 to 12 weeks old adult mice or from Sprague-Dawley rat pups at postnatal day 2 as previously described (Karch et al., 2019). MEFs at passages five to six or CFs at passages one to two were used for the experiments. To induce REV-ERB deletion, Cre Recombinase expressing adenovirus (Ad-Cre-GFP, with GFP reporter, Vector Biolabs, PA, United States) was transduced to cultured fibroblasts (MEFs and CFs) at a Multiplicity of Infection (MOI) rate of 400. The vector expressing GFP alone (Ad-GFP, Vector Biolabs, PA, United States) was used as the control. *Fibroblasts (MEFs and CFs) were cultured in DMEM* (*high glucose,* Thermo Fisher, with 10% fetal bovine serum, FBS), then switched to *low* serum condition (FBS, 0.1%) for 24 h prior to *activation*. *TGF-*β*1* (*10 mg/ml, R&D Systems, MN, United States*) *was added to the low*-serum *DMEM for 48hrs to induce the activation of fibroblasts to myofibroblasts.* The REV-ERB agonist SR9009 was given at a concentration of 10 μM when indicated.

### Immunofluorescence staining

Coverslips with fibroblasts culture were fixed with 100% methanol for 15 min. The cells were incubated with primary antibody against α-smooth muscle actin (ab5694, Abcam, 1:100 dilution) at 4°C overnight after blocking. Then, the cells were incubated with a fluorescence conjugated secondary antibody (A-11037, Invitrogen, 1:500 dilution) for 1 h in the dark and stained with DAPI (D3571, Invitrogen). Images were captured with EVOS FL Auto Imaging System (Life Technologies, Thermo Fisher Scientific, Inc.) and analyzed using ImageJ (National Institute of Health, United States).

### Reverse transcription and quantitative real-time PCR

Total RNA was extracted using a High Pure RNA Isolation Kit (Roche, Mannheim, Germany) according to the manufacturer’s protocol. The concentration was measured by a microplate reader (FLUOstar Omega, BMG LABTECH, Ortenberg, Germany). cDNA was synthesized using a reverse transcription supermix (iScript, BIO-RAD, CA, United States). Quantitative real-time PCR was performed on QuantStudio five Dx Real-Time PCR Systems (Applied Biosystems, Thermo Fisher Scientific, Inc.) with SYBR Green Supermix (SSO Advanced, BIO-RAD, CA, United States). All primers used in this manuscript are listed in Table 1. *Ppib* was used as a reference for normalization. The relative mRNA expression was calculated by the ΔΔCt method.

**TABLE 1:** Primers used for qRT-PCR.

Gene	Sequence
*Nr1d1* exon 4-F	CAT​GCC​AAC​GGA​GAG​ACA​CT
*Nr1d1* exon 4-R	GCC​GGA​GCA​TCC​AAC​AGA​AT
*Nr1d1*-F	ACG​ACC​CTG​GAC​TCC​AAT​AA
*Nr1d1*-R	CCA​TTG​GAG​CTG​TCA​CTG​TAG​A
*Nr1d2* exon 3-F	AGT​GGC​ATG​GTT​CTA​CTG​TGT
*Nr1d2* exon 3-R	GCC​TTC​ACA​AGC​ATG​AAC​TCC
*Nr1d2*-F	CAG​ACT​GAG​AAC​AGA​AAT​AGT​TAC​CTG
*Nr1d2*-R	GAG​ACT​TGC​TCA​TAG​GAC​ACA​CC
*Acta2*-F	ACT​GGG​ACG​ACA​TGG​AAA​AG
*Acta2*-R	GTT​CAG​TGG​TGC​CTC​TGT​CA
*Col1a1*-F	GCT​CCT​CTT​AGG​GGC​CAC​T
*Col1a1*-R	CCA​CGT​CTC​ACC​ATT​GGG​G
*Fn1*-F	ATG​TGG​ACC​CCT​CCT​GAT​AGT
*Fn1*-R	GCC​CAG​TGA​TTT​CAG​CAA​AGG
*Ppib*-F	TTC​TTC​ATA​ACC​ACA​GTC​AAG​ACC
*Ppib*-R	ACC​TTC​CGT​ACC​ACA​TCC​AT

### BrdU incorporation assay

The fibroblast DNA synthesis and proliferation was evaluated by BrdU Cell Proliferation Assay Kit (Cell Signaling Technology. Inc., MA, United States) following the manufacturer’s manual. Cells were incubated with BrdU for 6 h prior to the assay. The absorbance was measured at 450 nm by a microplate reader (FLUOstar Omega, BMG LABTECH, Ortenberg, Germany).

### Cell proliferation and cytotoxicity assay

The proliferation ability of CFs was assessed by Cell Counting Kit 8 (CCK-8, 96992-100TESTS-F Sigma) following the manufacturer’s manual. Cells were incubated with WST-8 (tetrazolium salt, Patent No. WO97/38985) Solution for 5 h prior to the assay.

### Wound healing assay

In order to study the dynamics of fibroblast activation during wound closure, CFs were seeded in monolayers and tested by the scratch assay as previously described (Liang et al., 2007). Images were acquired before and after scratch at multiple time points up to 36 h and analyzed by measuring the average distance of the gap by ImageJ (National Institute of Health, United States). For a single time point study, the residue wound is measured at a predetermined time point (18 h) and compared. For a multiple time point healing kinetics study, the cells were examined 5 times after the scratch wound for 24–36 h till complete closure of the wound.

### Immunoblotting

Cells were lysed in RIPA buffer supplemented with a complete protease inhibitor cocktail (Roche) and PhosSTOP (Roche). Lysates were resolved by gel electrophoresis (Bio-Rad), transferred to PVDF membranes (Immubilon-P, Millipore), and probed with the following antibodies: anti-REV-ERBα (1:500 (E1Y6D) Cell signaling, #13418), and anti-GAPDH (1:1,000, Cell signaling, #97166).

### Statistical analysis

Data was shown as means ± *SEM.* Comparisons were analyzed by Student’s t test, one-way ANOVA, or two-way analysis of variance (ANOVA). Multiple comparisons were taken into account when necessary. All statistical analysis was performed on IBM SPSS Statistics 22.0 (Armonk, NY, United States) or GraphPad Prism (San Diego, CA, United States). *p* < 0.05 was considered statistically significant. Experiments were repeated at least three times independently.

## Results

### Generation of the *Nr1d1/Nr1d2* double deletion MEFs model

**FIGURE 1:**
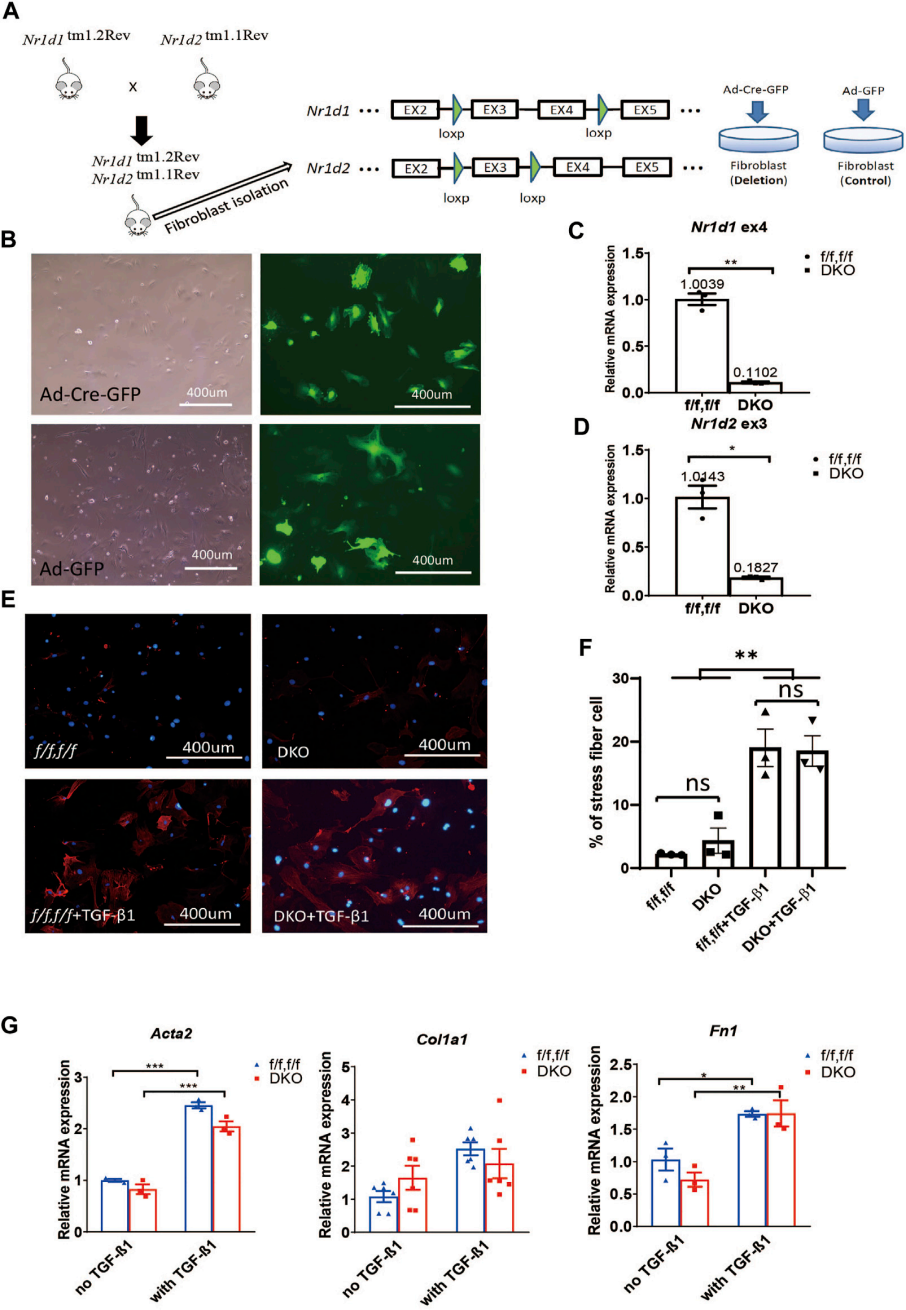
REV-ERB deletion does not affect MEFs activation. **(A)** Schematic illustration of the strategy to create fibroblasts (MEFs and CFs) models *in vitro*. *Nr1d1* ^tm1.2Rev^ and *Nr1d2* ^tm1.1Rev^ double floxed (*Nr1d1/2* ^*fl/fl*^) mouse line were generated by crossing. MEFs or CFs were isolated from double floxed mice, and REV-ERB was depleted by infection with Ad-Cre-GFP or Ad-GFP as control. Green triangle: loxP site. **(B)** Representative phase-contrast and GFP fluorescence images of MEFs from *Nr1d1/2* ^*fl/fl*^ mouse infected with Ad-Cre-GFP or Ad-GFP virus. **(C,D)** Relative mRNA expression of *Nr1d1* or *Nr1d2* with qRT-PCR primers spanning the loxP sites n = 3. **(E)** Representative images of immunostaining using α-SMA (Red) and DAPI (Blue) for *Nr1d1/2* ^*fl/fl*^ (control) and DKO MEFs with or without TGFβ-1 treatment. **(F)** Quantification of the percentage of the stress fiber positive cells in *Nr1d1/2* ^*fl/fl*^ (control) and DKO MEFs with or without TGFβ-1 treatment. Stress fiber was indicated by α-SMA staining. N = 3. **(G)** Relative mRNA expression of *Acta2*, *Col1a1,* and *Fn1* in *Nr1d1/2* ^*fl/fl*^ and DKO MEFs with or without TGFβ-1 treatment. *n*=3 to 6.*p = 0.05, ***p < 0.001, and ****p < 0.0001 by 2-sided Student’s t-test **(C,D)** and *P < 0.05, **P < 0.01 and ***P < 0.001 by two-way ANOVA **(F,G)**. Data are presented as mean ± SEM.

To study the REV-ERB function in the fibroblast, we isolated MEFs from the previously reported *Nr1d1* ^tm1.2Rev^ and *Nr1d2* ^tm1.1Rev^ double floxed mouse line (Song et al., 2022). We infected the MEFs with either Cre expressing (Ad-Cre-GFP) or control (Ad-GFP) adenovirus (Figure 1A). Percent GFP positive cells showed a near 100% infection efficiency. Cre induced deletion was validated by qRT-PCR, and the results showed an 89% reduction of *Nr1d1* and an 82% reduction of *Nr1d2* in the Ad-Cre-GFP infected MEFs (which we denoted as double knockout or DKO for simplicity) compared to the ad-GFP infected MEFs (double floxed controls, *Nr1d1/2* ^*fl/fl*^) (Figures 1B–D).

### Double deletion of *Nr1d1*/*Nr1d2* has no effect on MEFs activation

To examine the effect of REV-ERB on MEFs activation, we treated *Nr1d1/2* ^*fl/fl*^ and DKO MEFs with TGFβ-1, a known activator of MEFs *in vitro* (Leask, 2010; Dobaczewski et al., 2011). By immunostaining of the activated fibroblasts/myofibroblasts marker αSMA, we found there is a minimum signal of αSMA at the baseline for both the *Nr1d1/2* ^*fl/fl*^ and DKO MEFs. 48 h after treatment with TGFβ-1, both *Nr1d1/2* ^*fl/fl*^ and DKO MEFs showed similarly increased αSMA signals (Figures 1E,F). To further quantify the levels of fibroblasts activation in the TGFβ-1 treated *Nr1d1/2* ^*fl/fl*^ and DKO MEFs, we examined several myofibroblasts markers by qRT-PCR. The relative expression level of alpha smooth actin (*Acta2*), collagen I (*Col1a1*), and fibronectin (*Fn1*) were determined in *Nr1d1/2* ^*fl/fl*^ and DKO MEFs with or without TGFβ-1 treatment. Consistent with immunofluorescent staining of αSMA, the qRT-PCR of all three myofibroblasts markers showed a significant effect of TGFβ-1 in fibroblasts activation, but there is no difference between the *Nr1d1/2* ^*fl/fl*^ and DKO MEFs either in baseline or TGFβ-1 treated conditions (Figure 1G). Our results suggested that REV-ERB activity was not involved in MEFs activation upon TGFβ-1 stimulation.

### Double deletion of *Nr1d1*/*Nr1d2* enhances the CFs activation

**FIGURE 2:**
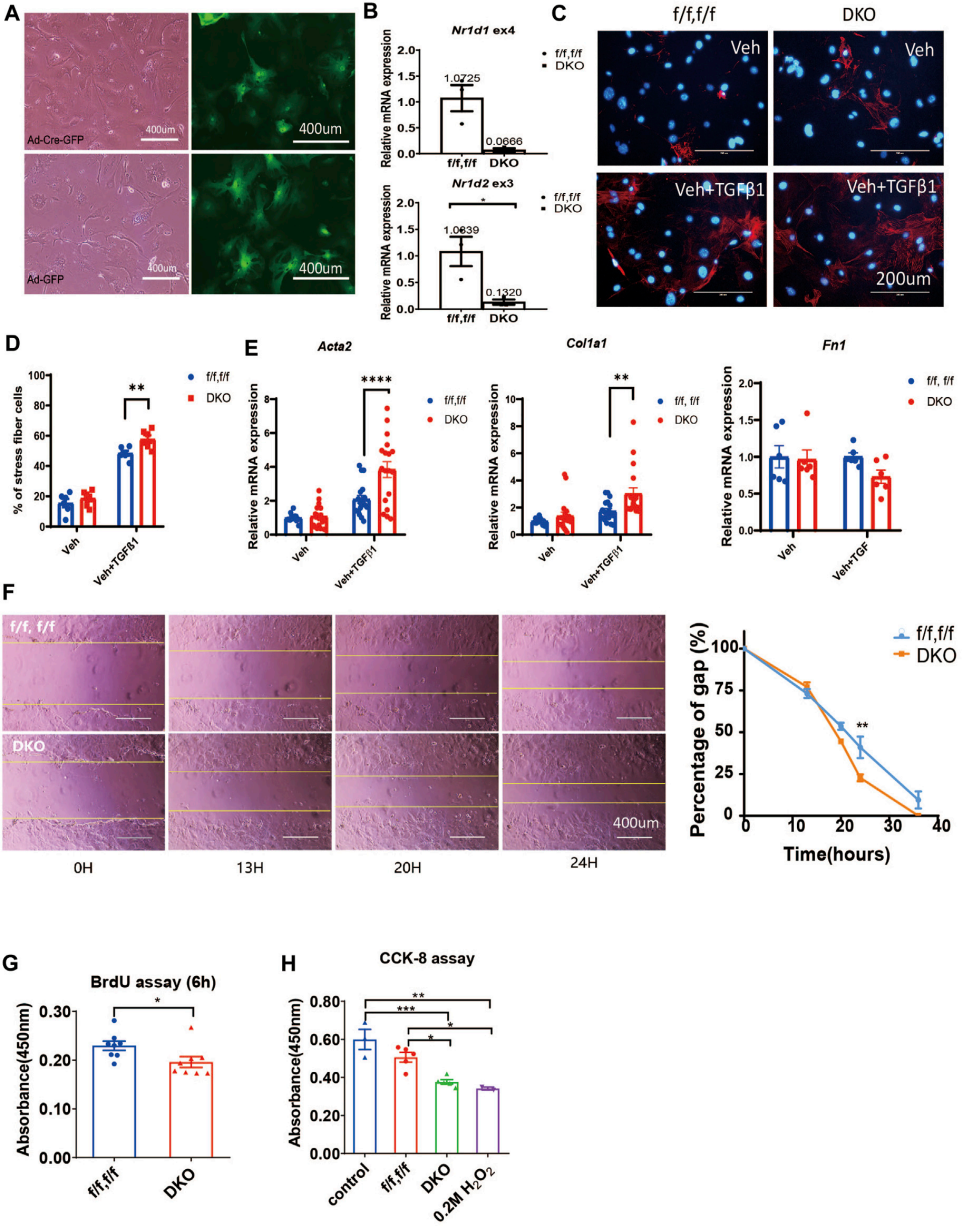
REV-ERB deletion leads to exaggerated activation of CFs. **(A)** Representative phase-contrast and GFP fluorescence images of CFs from *Nr1d1/2* ^*fl/fl*^ mouse infected with Ad-Cre-GFP or Ad-GFP virus. **(B)** Relative mRNA expression of *Nr1d1* and *Nr1d2* with qRT-PCR primers spanning loxP sites *n*=3. **(C)** Representative images of immunostaining of α-SMA (Red) and DAPI (Blue) for *Nr1d1/2* ^*fl/fl*^ (control) and DKO CFs with or without TGFβ-1 treatment. **(D)** Quantification of stress fiber positive cells in *Nr1d1/2* ^*fl/fl*^ (control) and DKO CFs with or without TGFβ-1 treatment *n*=6. **(E)** Relative mRNA expression of *Acta2*, *Col1a1,* and *Fn1* in *Nr1d1/2* ^*fl/fl*^ and DKO CFs with or without TGFβ-1 treatment *n*=6 to 18. **(F)** Representative images and quantification of the wound healing assay at different time points of the *Nr1d1/2* ^*fl/fl*^ and DKO CFs, n = 3, data are presented as mean ± SEM. **(G)** BrdU assay. The absorbance of BrdU incorporation was measured at 450 nm after 6 h incubation for *Nr1d1/2* ^*fl/fl*^ and DKO CFs, n = 8. **(H)** CCK-8 assay. The absorbance was measured at 450 nm for *Nr1d1/2* ^*fl/fl*^ and DKO CFs after 5h incubation with the WST-8 solution, n = 5. *p = 0.05, ***p < 0.001, and ****p < 0.0001 by one-way ANOVA **(H)**, two-way ANOVA **(D–F)**, and 2-sided Student’s t-test **(B and G)**. Data are presented as mean ± SEM.

Surprised by the results in MEFs, we went on to determine if REV-ERB plays a role in cardiac fibroblasts (CFs) activation. We isolated primary CFs from *Nr1d1/2* ^*fl/fl*^ mice and infected them with Cre expressing (Ad-Cre-GFP) or control (Ad-GFP) adenovirus. Infection efficiency was near 100%, and we found that the adenovirus induced Cre expression results in a 94% reduction of *Nr1d1* and an 87% reduction of *Nr1d2* in the ad-Cre-GFP infected CFs (Figures 2A,B). TGFβ-1 treatment was used to stimulate cardiac fibroblasts activation, immunostaining of the αSMA and qRT-PCR was performed on myofibroblasts markers. Interestingly, we found that while there is no difference at baseline, the DKO CFs showed higher αSMA signal positive cells compared to the control group with TGFβ-1 activation (Figures 2C,D). Consistent with this finding, the TGFβ-1 treatment induced higher expression of myofibroblasts markers *Acta2* and *Col1a1* but not *Fn1* in DKO CFs (Figure 2E).

We then further investigated the effect of REV-ERB on CF activation functionally, we performed wound healing, proliferation, and viability assays in the control *Nr1d1/2* ^*fl/fl*^ and DKO CFs. DKO CFs displayed a faster wound healing compared to the control *Nr1d1/2* ^*fl/fl*^ CFs with a significant difference at 24 h after the initial scratch, suggesting an increased proliferation, migration, and/or reduced death of the REV-ERB DKO CFs (Figure 2F). We then compared control *Nr1d1/2* ^*fl/fl*^ and DKO CFs in a BrdU incorporation assay to assess proliferation and in a CCK-8 assay for viability. Interestingly, DKO CFs showed both reduced proliferation and viability, which does not support faster wound-healing (Figures 2G,H). Taken together, we found DKO CFs showed reduced proliferation and viability but increased migration ability and increased fibroblast activation with TGFβ-1 treatment.

### SR9009 inhibits CFs activation

SR9009 has been shown to provide numerous health benefits in a variety of preclinical disease models *in vivo* (Zhang et al., 2017; Ishimaru et al., 2019; Reitz et al., 2019; Cunningham et al., 2020; Wolff et al., 2020). In particular, SR9009 offered cardioprotection in both pressure overload and ischemia-reperfusion models (Zhang et al., 2017; Reitz et al., 2019). Fibrosis was also significantly reduced by SR9009 post pressure overload (Zhang et al., 2017). However, whether the reduced cardiac fibrosis post pressure overload is a result of reduced cardiac injury or a direct effect of SR9009 on CFs remains unknown. Similarly, anti-fibrotic effect of SR9009 was observed in pulmonary and hepatic models (Cunningham et al., 2020; Griffett et al., 2020), suggesting that SR9009 might have a direct effect on fibrosis and that its anti-fibrotic effect in the heart may be independent of the myocyte injury.

**FIGURE 3:**
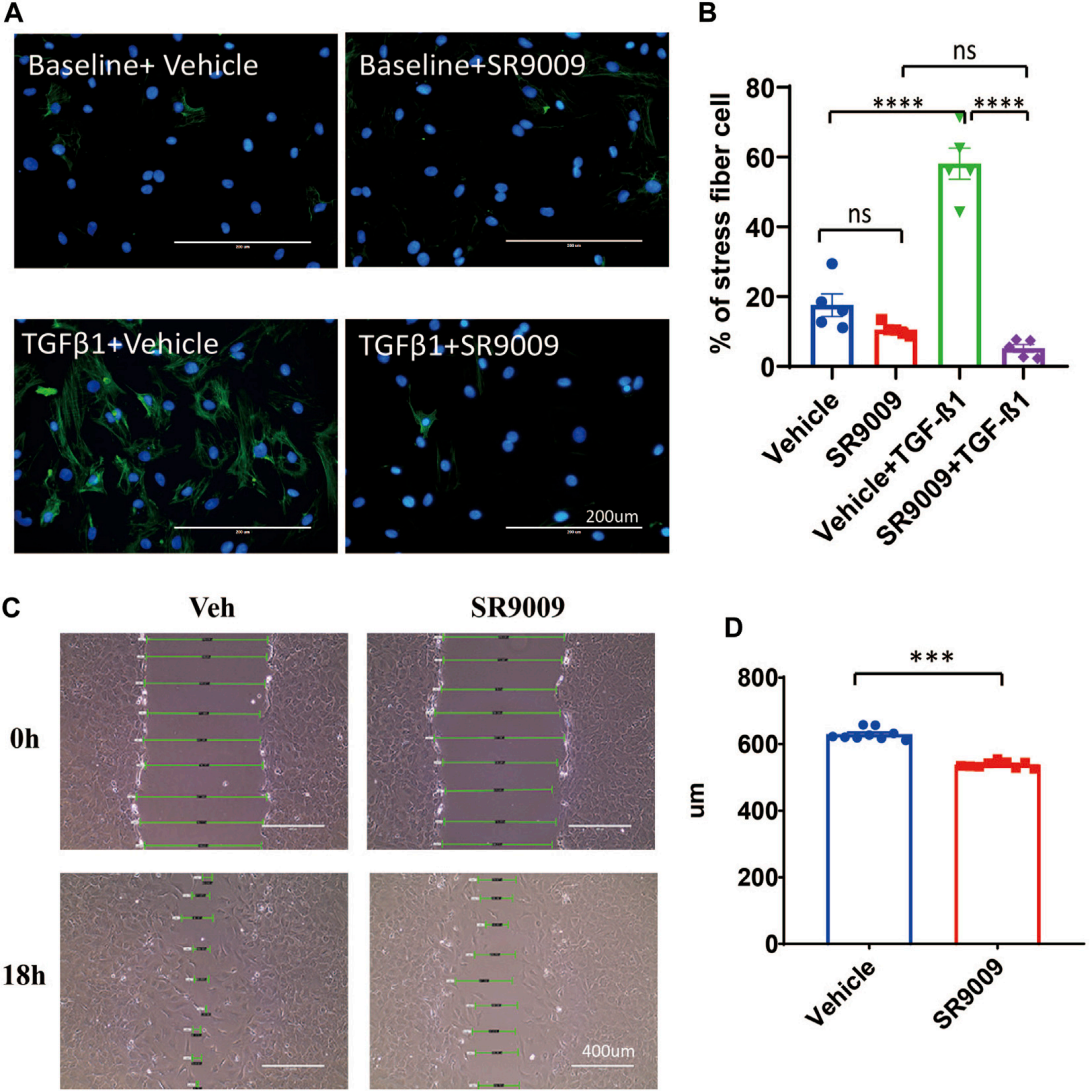
SR9009 inhibits CFs activation. **(A)** Representative immunostaining images showing α-SMA (Green) and DAPI (Blue) in vehicle or SR9009 treated CFs with or without TGFβ-1 treatment. **(B)** Quantification of the percentage of the stress fiber positive cells in vehicle or SR9009 treated CFs with or without TGFβ-1 treatment. Stress fiber was indicated by α-SMA staining, *n* = 5. **(C)** Representative images wound healing assay of CFs with or without SR9009 treatment at 0 and 18 h. **(D)** Quantification of the gap distance of the CFs treated with vehicle or SR9009 in the wound healing assay, *n* = 9. *p = 0.05, ***p < 0.001, and ****p < 0.0001 by one-way ANOVA **(B)** and 2-sided Student’s t-test **(D)**. Data are presented as mean ± SEM.

We treated the neonatal rat CFs with SR9009 in the presence or absence of TGFβ-1. We found that activation of CFs was completely blocked by SR9009 measured by immunostaining of αSMA (Figures 3A,B). Moreover, the SR9009-treated CFs showed a significantly impaired migration activity in a scratch assay (Figures 3C,D). This is consistent with the results that DKO CFs show increased activation and faster migration (Figure 2) suggesting SR9009 may work as an agonist of REV-ERB to inhibit CFs activation.

### SR9009 inhibits CFs and MEFs activation independent of REV-ERB

**FIGURE 4:**
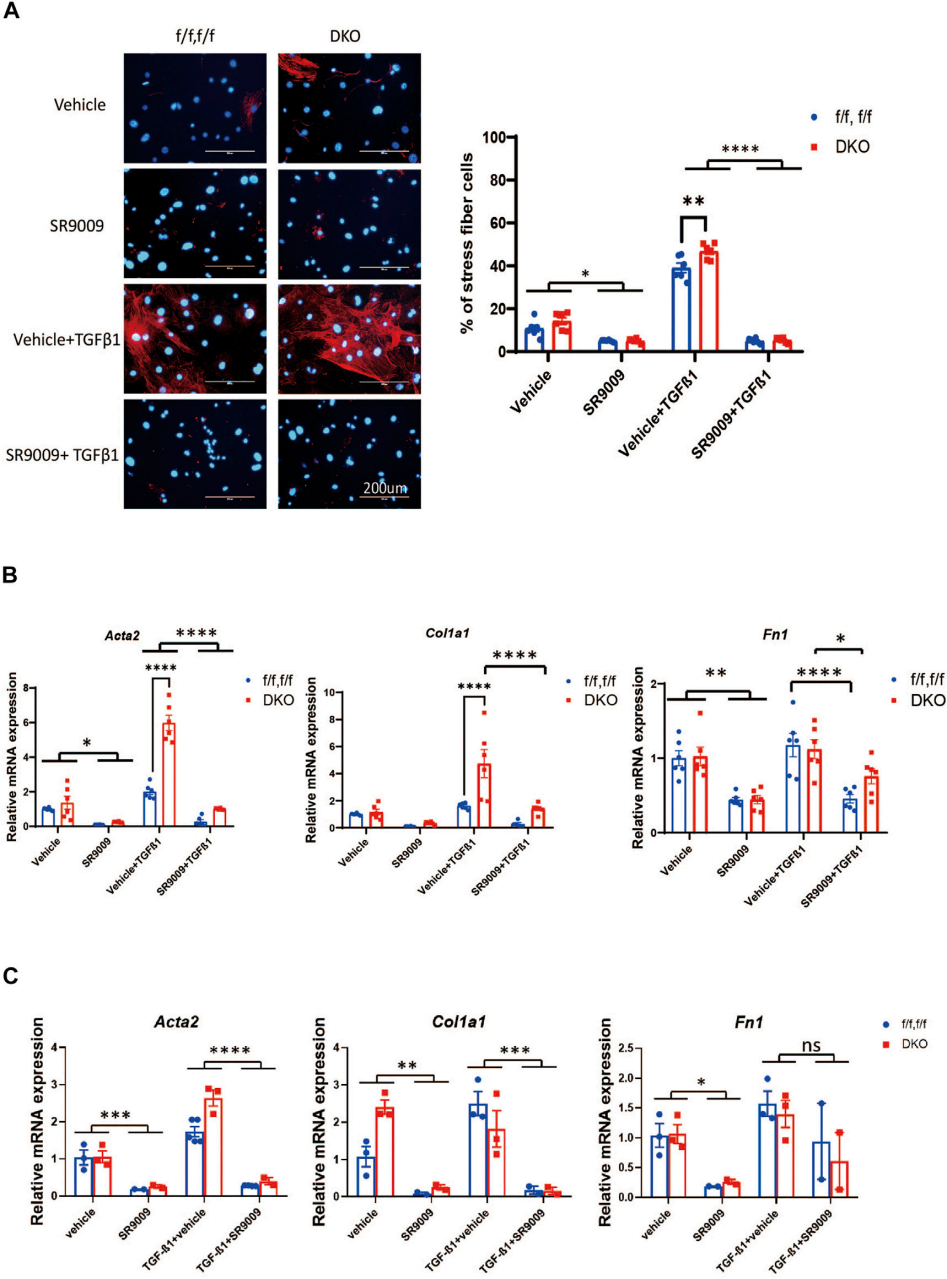
SR9009 inhibits MEFs activation independent of REV-ERB. **(A)** Representative images and quantification of immunostaining of α-SMA (Red) and DAPI (Blue) for *Nr1d1/2* ^*fl/fl*^ (control) and DKO CFs with or without SR9009 treatment and TGFβ-1 treatment *n*=6. **(B)** Relative mRNA expression of *Acta2*, *Col1a1*, and *Fn1* with or without SR9009 treatment in *Nr1d1/2* ^*fl/fl*^ and DKO CFs at baseline and post TGFβ-1 induced activation *n*=6. **(C)** Relative mRNA expression of *Acta2*, *Col1a1*, and *Fn1* with or without SR9009 treatment in *Nr1d1/2* ^*fl/fl*^ and DKO MEFs at baseline and post TGFβ-1 induced activation *n*=3. *p = 0.05, ***p < 0.001, and ****p < 0.0001 by two-way ANOVA, with multiple comparison corrected by Sidak, data are presented as mean ± SEM.

We then went on to test if the effect of SR9009 on fibroblasts is REV-ERB dependent. We treated *Nr1d1/2* ^*fl/fl*^ and DKO CFs or MEFs with or without TGFβ-1 and SR9009. Immunostaining of the αSMA was performed in CFs, interestingly, we found that SR9009 strongly suppressed the αSMA signals at baseline for both *Nr1d1/2* ^*fl/fl*^ and DKO CFs and most impressively, SR9009 completely blocked TGFβ-1 induced myofibroblasts activation for both genotype groups (Figure 4A). Similarly, qRT-PCR of myofibroblasts markers showed that SR9009 greatly reduced all myofibroblasts markers at baseline and abolished the activation upon TGFβ-1 stimulation in CFs of both genotypes (Figure 4B). Interestingly, this SR9009 mediated suppression of myofibroblasts activation was indistinguishable between *Nr1d1/2* ^*fl/fl*^ and DKO CFs (Figure 4B), A similar effect was also observed in *Nr1d1/2* ^*fl/fl*^ and DKO MEFs (Figure 4C), demonstrating that the SR9009 effect in inhibiting fibroblasts activation is independent of REV-ERB.

## Discussion

The cardiac protecting role of a circadian core clock factor, REV-ERB has been first established using a pharmacological tool drug, SR9009 (Zhang et al., 2017; Reitz et al., 2019). Although the target specificity of SR9009 has been challenged later, the role of REV-ERB in the heart was confirmed by more recent reports using the REV-ERB cardiac-specific knockout murine models (Dierickx et al., 2022; Song et al., 2022). REV-ERB has shown antifibrosis effects in the cardiac pressure-overload model as well as several other models, suggesting it may have an important role of REV-ERB in the cardiac fibroblasts (Zhang et al., 2017; Gonzalez-Fernandez et al., 2018; Cunningham et al., 2020; Griffett et al., 2020). In this study, we specifically interrogated the cell-autonomous role of REV-ERB in MEFs and CFs. Surprisingly, while REV-ERB does not seem to play any significant role in the MEF activation, it is critical in keeping CFs in quiescence. REV-ERB is essential for CF survival and proliferation. However, it inhibits CFs activation and wound healing. CFs with REV-ERBα/β double deletion showed increased activation upon TGFβ-1 treatment, reduced proliferation and viability, and faster wound healing. Since both viability and proliferation are reduced, the increased would healing is likely due to increased migration. Thus, REV-ERB seems to keep CFs in a healthy quiescent state.

Its function in CFs suggests that pharmacological targeting of REV-ERB simultaneuosly in both CFs and cardiomyocytes may lead to synergistic benefits in cardiac diseases. *In vivo* testing using CF-specific REV-ERB deletion models will be the next step to further validate this hypothesis.

Our study demonstrated that REV-ERBα/β play essential roles in CFs independent of cardiomyocytes and other cell types in the heart. This function likely contributes to the cardioprotective effect of REV-ERB agonist when administered systemically. Interestingly, we also found that REV-ERB does not carry a similar function in MEFs. There are many different types of fibroblasts residing in different tissue niches. In addition to their commonality, they may assume tissue-specific function and have unique responses to different stimuli. Dissecting out their differences will be an interesting future project, which will help understand the systemic consequences of pharmacological targeting of REV-ERB.

Another interesting observation is that SR9009 has a strong suppressive effect in CFs and MEFs activation that is independent of REV-ERB. Similar results were seen in both CFs and MEFs. Our results corroborated with previous reports and showed that SR9009 likely has off-target effects that contribute to its cardiac protective and anti-fibrotic functions (Dierickx et al., 2019; Li et al., 2022). A more detailed study to identify the possible mechanisms and targets of SR9009 is beyond the scope of the current study. Novel REV-ERB agonists with enhanced specificity will be needed to facilitate future studies of REV-ERB functions. These future novel compounds should be robustly tested in REV-ERB deleted cellular or animal models.

## Data Availability

The original contributions presented in the study are included in the article/supplementary material, further inquiries can be directed to the corresponding authors.
